# Longitudinal associations of internalized weight stigma with physical activity and weight loss

**DOI:** 10.1002/osp4.773

**Published:** 2024-07-03

**Authors:** Miriam Sheynblyum, Thomas A. Wadden, Janet D. Latner, Rebecca L. Pearl

**Affiliations:** ^1^ Department of Clinical and Health Psychology University of Florida Gainesville Florida USA; ^2^ Department of Psychiatry Perelman School of Medicine at the University of Pennsylvania Philadelphia Pennsylvania USA; ^3^ Department of Psychology University of Hawaii at Manoa Honolulu Hawaii USA

**Keywords:** exercise, obesity, stigma, weight loss

## Abstract

**Objective:**

Cross‐sectional research has demonstrated that internalized weight stigma (IWS) is associated with less engagement in weight management behaviors, including physical activity. However, limited research has explored longitudinal relationships among IWS, physical activity, and weight loss. This study examined longitudinal associations of changes in IWS, physical activity, and weight and tested whether physical activity mediated the relationship between IWS and weight change.

**Methods:**

Individuals with obesity and high IWS (*N* = 105) participated in a 72‐week behavioral weight loss intervention, with or without a weight stigma intervention. Measures of IWS (Weight Self‐Stigma Questionnaire), physical activity (accelerometry and self‐report), and weight were collected at baseline and weeks 20, 46 and 72. Correlations examined relationships among changes in variables from baseline to all timepoints, controlling for treatment condition. Mediation, controlling for treatment condition, tested whether IWS reductions during the first 20 weeks predicted greater weight loss at weeks 46 and 72 via increased physical activity between weeks 20 and 46 or 72.

**Results:**

Decreases in IWS at week 20 were associated with greater week‐20 weight loss (*r* = 0.265, *p* = 0.012). Physical activity was not a significant mediator, but greater reductions in IWS at week 20 predicted greater week‐46 weight loss with or without controlling for physical activity (WSSQ: *b* = 0.30, confidence interval: 0.12, 0.54). Significant associations were not found at week 72.

**Conclusion:**

Initial reductions in IWS were associated with greater week‐46 weight loss. Further research should investigate whether reducing IWS early in obesity treatment enhances long‐term outcomes.

**Clinical trial registration:**

ClinicalTrials.gov (NCT03704064).

## 1 INTRODUCTION

Weight stigma refers to the social devaluation of individuals with high body weight (e.g., obesity) and includes negative weight‐related attitudes (e.g., weight bias), stereotypes, and weight‐based discrimination.^1^ Internalization of weight stigma occurs when individuals agree with and apply negative weight‐based stereotypes to themselves, leading to self‐stigma.^2^ Internalized weight stigma (IWS) is associated with an array of adverse health outcomes, including greater depression and anxiety, lower self‐esteem, greater disordered eating, heightened cardiometabolic risk, and poorer health‐related quality of life.^3^

Relatively little research has examined how IWS relates to behavioral weight loss treatment and weight‐loss maintenance outcomes. Some studies have suggested that IWS may predict less weight loss and maintenance although the findings differ by race and gender.^4^, ^5^ There is also limited research examining the mechanisms that may contribute to the association between IWS and weight loss in a behavioral weight loss intervention.

Physical activity may serve as one such mechanism, given its critical role in facilitating and maintaining long‐term weight loss.^6^ Higher IWS is associated with less physical activity,^3^ which may in part be explained by reduced exercise self‐efficacy associated with internalizing negative weight stereotypes (e.g., laziness) and concerns about negative judgements from others while exercising.^7^, ^8^ Most investigations of IWS and physical activity have been cross‐sectional, with limited data on concurrent changes in IWS and physical activity. One study found that high IWS was associated with attenuated increases in physical activity during a healthy lifestyle program.^9^ More longitudinal research is needed to understand how IWS may impact physical activity and weight loss, and to determine whether *reducing* IWS may lead to increases in physical activity and enhanced long‐term weight loss outcomes.

The relationship between IWS and physical activity may be particularly important to examine among individuals who are attempting to maintain weight loss, given that physical activity is a key predictor of long‐term maintenance.^6^, ^10^ Weight loss maintenance is extremely challenging, and weight is regained at high rates.^10^ It is possible that individuals with lower IWS may be better able to navigate the challenges of weight loss maintenance with confidence, sticking to their physical activity goals and recovering from small weight gains with resilience. Weight loss is also inherently more rewarding than maintenance,^11^ and prior studies have shown at least modest short‐term reductions in IWS among individuals engaged in weight management.^9^, ^12^ However, no study to date has investigated whether initial reductions in IWS may predict subsequent engagement in physical activity and long‐term weight loss.

The current study examined longitudinal associations of IWS with physical activity and weight among 105 adults with obesity who were enrolled in a 72‐week behavioral weight loss intervention. Participants engaged in the most intensive part of the behavioral weight loss intervention during the first 20 weeks by attending weekly sessions focused on weight loss. To facilitate weight loss, the primary emphasis of this part of the intervention was dietary change (e.g., self‐monitoring of caloric intake), while participants were also encouraged to begin establishing a physical activity routine and increase their activity in small increments (up to 150 minutes per week). During the subsequent 52 weeks (weeks 20 through 72), the intervention primarily focused on weight loss maintenance, with less frequent (monthly and every‐other‐month) sessions. During the maintenance portion of the intervention, greater emphasis was placed on increasing physical activity (ultimately up to 250–300 min per week) while also encouraging participants to maintain their dietary habits. This approach aligns with recommendations stemming from evidence that increasing physical activity is particularly critical for maintaining long‐term weight loss.^10^, ^13^

The present study examined both concurrent and time‐lapsed associations of changes in IWS, physical activity, and weight. First, correlations among concurrent changes in IWS, physical activity, and weight were tested from baseline to weeks 20, 46, and 72 of the 72‐week behavioral weight loss intervention. Examining concurrent associations offers insights into how reducing IWS may simultaneously enhance changes in physical activity and weight (or vice versa, i.e., how increasing physical activity and/or losing weight may lead to reductions in IWS). It was hypothesized that decreases in IWS would be associated with concurrent increases in physical activity and greater weight loss at all time points. Additionally, this study examined whether changes in IWS from baseline to week 20 predicted total weight loss at weeks 46 and 72, and whether changes in physical activity from week 20 to weeks 46 and 72 mediated these relationships. This study hypothesized that greater initial decreases in IWS from baseline to week 20 would be associated with greater subsequent increases in participants' physical activity during the weight loss maintenance phase of the intervention, resulting in greater long‐term weight loss.

## 2 METHODS

This study was a secondary analysis of data from a randomized controlled trial that evaluated the effects of a psychological intervention designed to reduce IWS on long‐term weight loss in 105 adults with obesity.^12^ To be eligible for the trial, participants had to report at least one lifetime experience of weight stigma, high levels of IWS (defined as a score ≥4 on the Weight Bias Internalization Scale [WBIS] and confirmed by interview), and engaging in less than 150 min of structured physical activity per week. Individuals were excluded if they: had type I or II diabetes, uncontrolled hypertension, recent cardiovascular events, had lost and maintained ≥5% of their initial weight in the last 3 months or ≥10% in the past 2 years, were participating in psychotherapy related to weight, or had severe mood or binge eating disorder symptoms, bulimia nervosa, recent bariatric surgery, or pregnancy.^12^

### 2.1 Procedures

Participants in the trial were randomized to receive either behavioral weight loss treatment alone or behavioral weight loss combined with a weight stigma‐reduction intervention. The behavioral weight loss intervention for both conditions consisted of 20 weekly, 60‐min group sessions focused on weight loss, followed by 6 monthly and 3 every‐other‐month weight‐loss maintenance sessions delivered across 52 weeks (72 weeks total). Starting at week 5, half of the participants received the weight stigma‐reduction intervention for an additional 30 minutes during each behavioral weight loss session, while the other half spent this time discussing recipes and cooking tips. Due to minimal significant between‐group differences for most outcomes,^12^ data were combined across conditions in this secondary analysis, with treatment condition included as a covariate in all analyses. Participants were assessed at baseline and at weeks 20, 46 and 72.

### 2.2 Measures

#### 2.2.1 Internalized weight stigma

For the current study, the primary measure of IWS was the Weight Self‐Stigma Questionnaire (WSSQ).^14^ The WSSQ consists of 12 items rated on a 5‐point scale and summed to generate a total score ranging from 12 to 60. Items assess self‐devaluation (e.g., “I became overweight because I am a weak person”) and concerns about stigmatization by others (e.g., “People think that I am to blame for my weight problems”). This measure has strong psychometric properties, with Cronbach's *α* ranging from 0.79 to 0.84 across the four time points in the current study.^14^

Internalized weight stigma was also assessed using the 10‐item WBIS, with ratings on a 7‐point scale.^2^, ^15^ The WBIS has strong psychometric properties and is a commonly used measure of IWS.^2^, ^3^ Items address agreement with weight‐related stereotypes (e.g., “I am less attractive than most other people because of my weight”) and self‐devaluation (e.g., “My weight is a major way that I judge my value as a person”). The WBIS demonstrated good internal consistency in the current sample across the four time points (Cronbach's *α* ranged from 0.74 to 0.84). Because the WBIS was used to determine eligibility for the study, all participants had a score of ≥4. Due to the restricted score range, this secondary analysis focused only on the WSSQ. Supplemental analyses with the WBIS are found in Tables S1‐S7.

#### 2.2.2 Physical activity

Physical activity was measured using both objective and self‐report methods, aligning with research recommendations for the complementary use of objective assessment alongside self‐report measures.^16^ To assess physical activity objectively, participants were instructed to wear triaxial accelerometers (ActiGraph GT9X) on their wrists for 8 days at each time point (baseline, weeks 20, 46, and 72), and data from the most recent full 7 days were used for analyses, with wear‐time validation requiring a minimum of 600 minutes of wear per day. Participants with at least 4 days of data (3 weekdays and 1 weekend day) were included in analyses, and data were averaged across the number of complete days. Accelerometry assessed two aspects of physical activity: (1) minutes per day spent engaging in total moderate‐intensity physical activity (i.e., *unbouted* activity), and (2) minutes per day spent engaging in moderate‐to‐vigorous physical activity (MVPA) with activity bouts lasting at least 20 minutes (referred to here as *bouted MVPA*). Intensity thresholds were based on established activity counts.^17^

Self‐reported structured physical activity was measured by interview using the Paffenbarger Exercise Habits Questionnaire.^18^ The Compendium of Physical Activities was used to compute estimates of energy expenditure over the last week, presented as kilocalories (kcal).^19^ The Compendium lists various forms of physical activity and their corresponding metabolic equivalent of task (MET) values.^19^ Participants' energy expenditure was estimated by multiplying the MET value by the times per week and duration that individuals engaged in each activity.^18^

#### 2.2.3 Weight

Weight was measured in duplicate using a digital clinic scale (Detecto, Model 6800A). Due to the COVID‐19 pandemic, some weight measurements were obtained remotely at weeks 20, 46, and 72 with an EatSmart Precision Digital Scale.^12^ Remote weights were measured in duplicate and documented by participants with photographs. Measurements taken with at‐home scales were validated against clinic scale measurements after COVID‐19 restrictions were lifted.^12^ Percentage of body weight change was computed from baseline to weeks 20, 46, and 72, respectively. A dichotomous variable was also created to indicate whether participants had a weight loss of ≥5% from baseline to weeks 46 and 72; this threshold is associated with clinically meaningful cardiometabolic health improvements.^20^

### 2.3 Statistical analyses

Data were inspected for missing and out of range values. Accelerometry data from two participants were excluded due to implausibly high values. Continuous measures were assessed for normality. At all time points, a square root transformation was used for self‐reported energy expenditure, and a logarithmic transformation was used for bouted MVPA. All other measures were normally distributed.

#### 2.3.1 Correlations

To test if changes in IWS were associated with concurrent changes in physical activity and weight, partial correlations using complete data and controlling for treatment condition, tested associations between changes in IWS, physical activity (unbouted moderate‐intensity physical activity, bouted MVPA, and self‐reported energy expenditure), and weight from baseline to weeks 20, 46, and 72, respectively.

#### 2.3.2 Primary mediation analyses: Baseline to week 46

**FIGURE 1:**
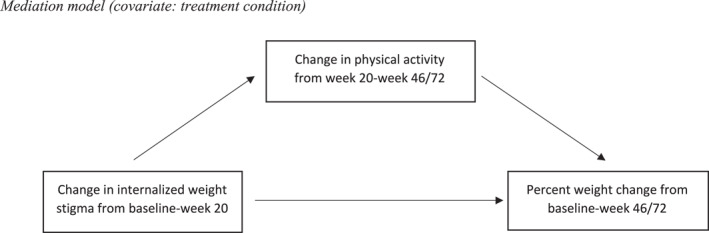
Mediation model (covariate: treatment condition).

Bootstrapping mediation analyses tested change in physical activity as a mediator between week‐20 changes in IWS and percent weight change at week 46, with group condition included as a covariate (see Figure 1). The SPSS macro provided by Preacher and Hayes^21^ was used to estimate both direct and indirect effects based on 1000 bootstrap samples and a 95% confidence interval (CI), with the significance level of *p* < 0.05. Analyses were conducted using last observation carried forward (LOCF) to account for missing data. Analyses tested whether change in physical activity from week 20 to 46 mediated the relationship between change in IWS at week 20 and percent weight loss (Model 1) or odds of ≥5% weight loss (Model 2) from baseline to week 46. Models were initially tested using unbouted moderate‐intensity physical activity as the mediator, and then repeated with bouted MVPA and self‐reported weekly energy expenditure. Treatment conditions were controlled for in all mediation models.

#### 2.3.3 Primary mediation analyses: Baseline to week 72

Next, analyses examined whether change in physical activity from week 20 to week 72 mediated the relationship between change in IWS from baseline to week 20 and percent weight change (Model 3) or odds of ≥5% weight loss (Model 4) from baseline to week 72. Treatment condition was controlled for in all mediation models.

#### 2.3.4 Sensitivity mediation analyses with Completer's analysis

Models 1–4 described above were conducted using complete data as a sensitivity mediation analysis.

SPSS 28.0 was used in all analyses. The trial was approved by the institutional review boards at the University of Pennsylvania and the University of Florida, and all participants provided written informed consent.

## 3 RESULTS

### 3.1 Participant characteristics

Participants were predominantly white women (70.5% white, 24.5% Black; 90.5% women) of middle age (*M* = 49.06 ± 12.40 years), with a mean body mass index (BMI) of 37.95 ± 5.54 kg/m^2^ (Table 1). Descriptively, average change in WSSQ scores from baseline to week 72 was *M*_Δ_ = −8.29 ± 8.78 on a scale from 12 to 60. Average changes in physical activity variables from baseline to week 72 were as follows: self‐reported weekly energy expenditure: *M*_Δ_ = 390.05 ± 987.51 kcal/week; bouted MVPA:*M*_Δ_ = 8.87 ± 18.66 min/d; unbouted moderate‐intensity physical activity: *M*_Δ_ = −2.63 ± 45.54 min/d. Average percent weight change from baseline to week 72 was −5.84 ± 8.16% (See Tables 2, 3, 4 footnotes for average changes in variables at other time points).

**TABLE 1:** Participant characteristics at baseline: (*N* = 105).

Variable	*N* (%) or mean ± standard deviation
Age (years)	49.06 ± 12.40
Gender	
Male	10 (9.5%)
Female	95 (90.5%)
Race	
American Indian or Alaska native	1 (1.0%)
Asian	1 (1.0%)
Black/African American	26 (24.8%)
Multiracial	3 (2.9%)
White	74 (70.5%)
Ethnicity	
Hispanic or Latino	3 (2.9%)
Not Hispanic or Latino	102 (97.1%)
Education	
Less than 4‐year college	36 (34.3%)
Four‐year college or more	69 (65.7%)
Body mass index (kg/m^2^)	37.95 ± 5.54

**TABLE 2:** Partial correlations: Baseline‐week 20.

Variable (change from baseline‐week 20)	1	2	3	4
1. Weight self‐stigma questionnaire (WSSQ)				
2. Percent weight change	0.265*			
3. Energy expenditure (kcal/week)^a^	0.090	0.035		
4. Unbouted moderate physical activity per day (minutes/day)	−0.076	−0.151	0.207	
5. Bouted moderate‐to‐vigorous physical activity minutes per day (MVPA; 20‐min bouts)^b^	−0.057	−0.096	0.484**	0.329**

*Note*: Sample sizes for correlations were as follows: Variable 1: *n* = 92; Variable 2: *n* = 90; Variable 3: *n* = 88; Variable 4: *n* = 76; Variable 5: *n* = 76. Partial correlations control for treatment condition. Average change values for untransformed variables (Mean ± Standard Deviation) were as follows: WSSQ = −6.80 ± 7.17; Percent Weight Change = −6.07 ± 4.65; Energy Expenditure = 389.07 ± 859.14 kcal/week; Unbouted moderate physical activity = 9.13 ± 34.41 min/d; Bouted MVPA = 10.21 ± 16.23 min/d.

^a^ Variable was transformed using the square‐root method.

^b^ Variable was transformed using the base‐10 logarithm.

*p < 0.05; **indicates *p* < 0.01.

**TABLE 3:** Partial correlations: Baseline‐week 46.

Variable (change from baseline‐week 46)	1	2	3	4
1. Weight self‐stigma questionnaire (WSSQ)				
2. Percent weight change	0.167			
3. Energy expenditure (kcal/week)^a^	−0.168	−0.122		
4. Unbouted moderate physical activity per day (minutes/day)	−0.077	−0.172	0.058	
5. Bouted moderate‐to‐vigorous physical activity minutes per day (MVPA; 20‐min bouts)^b^	−0.338**	−0.201	0.292*	0.294*

*Note*: Note. Sample sizes for correlations were as follows: Variable 1: *n* = 89; Variable 2: *n* = 90; Variable 3: *n* = 88; Variable 4: *n* = 59; Variable 5: *n* = 59. Partial correlations control for treatment condition. Average change values for untransformed variables (Mean ± Standard Deviation) were as follows: WSSQ = −6.94 ± 8.23; Percent Weight Change = −6.73 ± 6.93; Energy Expenditure = 186.55 ± 856.28 kcal/week; Unbouted moderate physical activity = −9.58 ± 43.88 min/d; Bouted MVPA = 10.19 ± 18.60 min/d.

^a^ Variable was transformed using the square‐root method.

^b^ Variable was transformed using the base‐10 logarithm.

*p < 0.05; **indicates *p* < 0.01.

**TABLE 4:** Partial correlations: Baseline‐week 72.

Variable (change from baseline‐week 72)	1	2	3	4
1. Weight self‐stigma questionnaire (WSSQ)				
2. Percent weight change	0.159			
3. Energy expenditure (kcal/week)^a^	0.061	−0.100		
4. Unbouted moderate physical activity per day (minutes/day)	−0.032	−0.295*	−0.098	
5. Bouted moderate‐to‐vigorous physical activity minutes per day (MVPA; 20‐min bouts)^b^	−0.053	−0.230	0.033	0.308**

*Note*: Sample sizes for correlations were as follows: Variable 1: *n* = 85; Variable 2: *n* = 89; Variable 3: *n* = 83; Variable 4: *n* = 71; Variable 5: *n* = 71. Partial correlations control for treatment condition. Average change values for untransformed variables (Mean ± Standard Deviation) were as follows: WSSQ = −8.29 ± 8.78; Percent Weight Change = −5.84 ± 8.16; Energy Expenditure = 390.05 ± 987.51 kcal/week; Unbouted moderate physical activity = −2.63 ± 45.54 min/d; Bouted MVPA = 8.87 ± 18.66 min/d.

^a^ Variable was transformed using the square‐root method.

^b^ Variable was transformed using the base‐10 logarithm.

*p < 0.05; **p < 0.01.

### 3.2 Correlations

From baseline to week 20 (Table 2), greater decreases in WSSQ scores were associated with greater percent weight loss (*r* = 0.265, *p* = 0.012). No other significant correlations from baseline to week 20 were observed.

From baseline to week 46 (Table 3), no associations were found between IWS and weight change. Greater decreases in WSSQ scores were associated with greater increases in bouted MVPA (*r* = −0.338, *p* = 0.009), but not the other physical activity variables (unbouted moderate‐intensity physical activity: *r* = −0.077, *p* = 0.565; self‐reported energy expenditure: *r* = −0.168, *p* = 0.120). No other significant correlations were found among variables from baseline to week 46.

From baseline to week 72 (Table 4), no associations were found between changes in IWS and changes in weight or physical activity. Greater increases in unbouted moderate‐intensity physical activity were associated with greater week‐72 percent weight loss (*r* = −0.295, *p* = 0.013). No other significant correlations from baseline to week 72 were observed.

### 3.3 Mediation analyses

#### 3.3.1 Primary mediation analyses: Baseline to week 46

In LOCF analyses (Table 5), change in physical activity from week 20‐46 did not mediate the relationship between change in WSSQ scores at week 20 and percent weight change (Table 5, Model 1) or odds of ≥5% weight loss (Table 5, Model 2) at week 46. However, a decrease in WSSQ scores at week 20 was associated with greater week‐46 percent weight loss (*b* = 0.30, *SE* = 0.10, 95% bootstrap CI: 0.12, 0.54) and higher odds of obtaining ≥5% weight loss (*b* = −0.11, *SE* = 0.04, 95% bootstrap CI: −0.20, −0.05), while controlling for unbouted moderate‐intensity physical activity. These findings were consistent when the models controlled for bouted MVPA or self‐reported energy expenditure (Table 5). These findings were also significant when physical activity was not included in the model (total effect: *b* = 0.30 *SE* = 0.09, 95% bootstrap CI: 0.002, 0.11).

**TABLE 5:** Bootstrap mediation results: LOCF analysis from Baseline to Week 46.

1	Unbouted	IWS ‐> unbouted moderate physical activity (*a*)	0.14	0.67	−1.08, 1.48
	Moderate physical activity	Unbouted moderate physical activity ‐> percent weight change (*b*)	0.00	0.02	−0.03, 0.04
		Direct effect of IWS ‐> percent weight change (*c’*)	**0.30**	**0.10**	**0.12, 0.54**
		Total effect of IWS ‐> percent weight change (*c*)	**0.30**	**0.09**	**0.002, 0.11**
		Indirect effect (*a × b*)	0.00	0.01	−0.03, 0.02
	Bouted MVPA	IWS ‐> bouted MVPA (*a*)	−0.01	0.00	−0.02, 0.00
		Bouted MVPA ‐> percent weight change (*b*)	−1.24	2.03	−6.18, 2.51
		Direct effect of IWS ‐> percent weight change (*c’*)	**0.29**	**0.10**	**0.12, 0.52**
		Total effect of IWS ‐> percent weight change (*c*)	**0.30**	**0.09**	**0.002, 0.11**
		Indirect effect (*a × b*)	0.01	0.02	−0.02, 0.05
	Energy	IWS ‐> energy expenditure (*a*)	−17.47	12.09	−42.54, 4.80
	Expenditure	Energy expenditure ‐> percent weight change (*b*)	0.00	0.00	0.00, 0.00
		Direct effect of IWS ‐> percent weight change (*c’*)	**0.30**	**0.09**	**0.13, 0.51**
		Total effect of IWS ‐> percent weight change (*c*)	**0.29**	**0.09**	**0.002, 0.10**
		Indirect effect (*a × b*)	−0.02	0.02	−0.08, 0.01
2	Unbouted	IWS ‐> unbouted moderate physical activity (*a*)	0.14	0.67	−1.01, 1.58
	Moderate physical activity	Unbouted moderate physical activity ‐> odds of ≥5% weight loss (*b*)	0.01	0.01	−0.01, 0.02
		Direct effect of IWS ‐> odds of ≥5% weight loss (*c’*)	**−0.11**	**0.04**	**−0.20, −0.05**
		Indirect effect (*a × b*)	0.00	0.01	−0.01, 0.02
	Bouted MVPA	IWS ‐> bouted MVPA (*a*)	−0.01	0.00	−0.02, 0.00
		Bouted MVPA ‐> odds of ≥5% weight loss (*b*)	0.11	0.74	−1.34, 1.74
		Direct effect of IWS ‐> odds of ≥5% weight loss (*c’*)	**−0.10**	**0.04**	**−0.29, −0.04**
		Indirect effect (*a × b*)	0.00	0.01	−0.02, 0.01
	Energy	IWS ‐> energy expenditure (*a*)	−17.47	12.22	−42.05, 6.80
	Expenditure	Energy expenditure ‐> odds of ≥5% weight loss (*b*)	0.00	0.00	0.00, 0.00
		Direct effect of IWS ‐> odds of ≥5% weight loss (*c’*)	**−0.11**	**0.04**	**−0.20, −0.05**
		Indirect effect (*a × b*)	0.00	0.01	−0.01, 0.02

*Note*: Predictor: change in IWS from baseline to week 20; Mediator: change in physical activity from week 20 to 46; Outcome: percent weight loss (Model 3) or odds of ≥5% weight loss (Model 4) from baseline to week 46. This table contains unstandardised coefficients. Confidence intervals that do not contain zero indicate a significant model and are highlighted in bold.

Abbreviations: IWS, internalized weight stigma; MVPA, moderate‐to‐vigorous physical activity; WSSQ, weight self‐stigma questionnaire.

#### 3.3.2 Primary mediation analyses: Baseline to week 72

In LOCF analyses (Table 6), change in physical activity from week 20–72 (measured via accelerometry or self‐report) did not mediate the relationship between change in WSSQ scores at week 20 and percent weight change (Table 6, Model 3) or odds of ≥5% weight loss (Table 6, Model 4) at week 72. There was a significant direct effect of IWS on percent weight change, but only when bouted MVPA was controlled for in the model (*b* = 0.21, *SE* = 0.12, 95% bootstrap CI: 0.01, 0.47), and not when controlling for the other physical activity variables.

**TABLE 6:** Bootstrap mediation results: LOCF analysis from Baseline to Week 72.

Model	Mediator	Path	Estimate	SE	CI
3	Unbouted	IWS ‐> unbouted moderate physical activity (*a*)	−0.14	0.53	−1.21, 0.89
	Moderate physical activity	Unbouted moderate physical activity ‐> percent weight change (*b*)	**−0.03**	**0.02**	**−0.07, −0.001**
		Direct effect of IWS ‐> percent weight change (*c’*)	0.20	0.12	−0.02, 0.45
		Total effect of IWS ‐> percent weight change (*c*)	0.19	0.11	0.07, −0.02
		Indirect effect (*a × b*)	0.00	0.02	−0.03, 0.04
	Bouted MVPA	IWS ‐> bouted MVPA (*a*)	0.01	0.01	−0.01, 0.03
		Bouted MVPA ‐> percent weight change (*b*)	−1.13	1.23	−3.61, 1.18
		Direct effect of IWS ‐> percent weight change (*c’*)	**0.21**	**0.12**	**0.01, 0.47**
		Total effect of IWS ‐> percent weight change (*c*)	0.20	0.11	0.08, −0.02
		Indirect effect (*a × b*)	−0.01	0.02	−0.07, 0.01
	Energy	IWS ‐> energy expenditure (*a*)	7.51	12.84	−19.07, 32.31
	Expenditure	Energy expenditure ‐> percent weight change (*b*)	0.00	0.00	0.00, 0.00
		Direct effect of IWS ‐> percent weight change (*c’*)	0.18	0.11	−0.01, 0.42
		Total effect of IWS ‐> percent weight change (*c*)	0.18	0.11	0.10, −0.03
		Indirect effect (*a × b*)	0.00	0.01	−0.04, 0.02
4	Unbouted	IWS ‐> unbouted moderate physical activity (*a*)	−0.14	0.54	−1.27, 0.86
	Moderate physical activity	Unbouted moderate physical activity ‐> odds of ≥5% weight loss (*b*)	0.01	0.01	0.00, 0.02
		Direct effect of IWS ‐> odds of ≥5% weight loss (*c’*)	−0.05	0.04	−0.13, 0.01
		Indirect effect (*a × b*)	0.00	0.01	−0.02, 0.01
	Bouted MVPA	IWS ‐> bouted MVPA (*a*)	0.01	0.01	−0.01, 0.03
		Bouted MVPA ‐> odds of ≥5% weight loss (*b*)	0.51	0.38	−0.16, 1.29
		Direct effect of IWS ‐> odds of ≥5% weight loss (*c’*)	−0.05	0.04	−0.13, 0.01
		Indirect effect (*a × b*)	0.00	0.01	−0.01, 0.02
	Energy	IWS ‐> energy expenditure (*a*)	7.51	12.87	−19.03, 32.58
	Expenditure	Energy expenditure ‐> odds of ≥5% weight loss (*b*)	0.00	0.00	0.00, 0.00
		Direct effect of IWS ‐> odds of ≥5% weight loss (*c’*)	−0.05	0.04	−0.14, 0.00
		Indirect effect (*a × b*)	0.00	0.00	−0.01, 0.01

*Note*: Predictor: change in IWS from baseline to week 20; Mediator: change in physical activity from week 20 to 72; Outcome: percent weight loss (Model 3) or odds of ≥5% weight loss (Model 4) from baseline to week 72. This table contains unstandardised coefficients, Confidence intervals that do not contain zero indicate a significant model and are highlighted in bold.

Abbreviations: IWS, internalized weight stigma; MVPA, moderate‐to‐vigorous physical activity; WSSQ, Weight Self‐Stigma Questionnaire.

#### 3.3.3 Sensitivity mediation analyses with Completer's analysis

In sensitivity mediation analyses that used complete data instead of LOCF, no significant associations of IWS with physical activity or weight loss were observed (Table S8).

## 4 DISCUSSION

This study is the first to test longitudinal associations among changes in IWS, physical activity, and weight among individuals with high IWS enrolled in a behavioral weight loss intervention. In partial support of this study's hypothesis, correlation results demonstrated that a decrease in IWS (measured by the WSSQ) from baseline to week 20 was associated with greater percent weight loss at week 20. However, this association was not found for other time points. In addition, a greater decrease in IWS at week 46 was associated with a simultaneous increase in time spent engaging in bouts of 20‐minutes or more of MVPA per day. This finding aligns with previous cross‐sectional research showing negative associations between IWS and physical activity.^22^ Notably, this effect was not observed at other time points or with other metrics of physical activity.

Physical activity was not a significant mediator in the relationship between early changes in IWS and weight change at weeks 46 and 72 of the intervention. However, with or without controlling for physical activity, greater decreases in IWS from baseline to week 20 predicted greater week‐46 percent weight loss and odds of losing ≥5% of initial body weight. This relationship between IWS and weight loss at week 46 is consistent with evidence from prior studies that lower IWS predicts greater weight loss and weight loss maintenance at one year.^4^, ^23^ Of note, sensitivity analyses with complete data did not show a significant relationship between IWS and week‐46 weight loss. Due to challenges related to the COVID‐19 pandemic, there was a higher degree of missing data for some physical activity variables at week 46 compared with other time points,^12^ which may in part explain this discrepancy in findings.

The current study also found that greater decreases in WSSQ scores at week 20 predicted greater week‐72 percent weight loss, specifically when bouted MVPA (and not other physical activity variables) was included in the model. Given that this effect was only significant with select variables, it requires caution when interpreting. Replication is needed to provide greater clarity on the effects of early reductions in IWS in long‐term weight loss. It is also possible that other unmeasured variables beyond physical activity may potentially explain or facilitate a relationship between early reductions in IWS and weight loss outcomes. For example, eating is a common coping response to the stress of stigma^24^ and could affect factors relevant to weight management such as food choices and portion sizes. Future studies could test whether reducing IWS predicts greater improvements in nutrient quality of food choices and adherence to dietary goals, which may contribute to greater weight loss.

Strengths of the current study include the use of both objective and self‐reported measures of physical activity. Additionally, this is one of the very few studies to report long‐term changes in IWS and weight‐related treatment outcomes, contributing to a literature that has largely relied on cross‐sectional and retrospective recall data.

This study also had several limitations. All findings presented in this report were based on secondary observational analyses, and no conclusions can be drawn about causality. The sample also contained missing data, particularly for the accelerometry‐based physical activity variables due to obstacles faced during the COVID‐19 pandemic.^12^ This study used LOCF as a conservative approach to account for missing data in mediation analyses, with completer's analysis utilised as a sensitivity analysis. Due to the inconsistency in findings between LOCF and completer's analyses, and the limitations of LOCF analysis, it is important to note that other methods of handling missing data might have produced different results. Moreover, major disruptions to normal life during the pandemic might have impacted individuals' activity levels and other weight‐related factors that influenced the results. Replication of this work in a larger sample and during a post‐pandemic period is needed.

Additionally, this study's sample primarily consisted of women, which may limit the generalisability of these findings to men and individuals from gender, racial, and ethnic minority populations. This study did not examine whether sociodemographic characteristics may predict or moderate the observed relationships, which requires future research with larger and more diverse samples. Multiple analyses were conducted in this study ‐ including separate analyses for each of the three physical activity variables and completer's analyses for sensitivity – which could have increased type 1 error. Additionally, this study had a relatively small sample size, which may have resulted in limited power and increased the risk of type 2 error. Given the lack of longitudinal data assessing relationships among IWS, physical activity, and weight, this preliminary work needs to be replicated in future studies to increase confidence in the findings.

With further investigation and replication, these findings may have implications for targeting IWS in clinical settings, including in the context of obesity treatment. Future research can test strategies to reduce IWS in the early stages or before initiation of behavioral weight loss treatment and assess the potential influence on long‐term weight‐related outcomes. In the current trial, the initial month of treatment in both conditions was focused exclusively on behavioral weight loss treatment, and the stigma intervention did not begin until after participants had an opportunity to master core behavioral skills such as dietary monitoring and building a physical activity routine.^12^ A study that randomizes the order in which participants receive a stigma versus behavioral weight loss intervention could elucidate whether targeting IWS early improves treatment outcomes. In addition, although the current study was focused on weight loss outcomes, more research is needed to understand other possible long‐term benefits for mental and physical health of reducing IWS among individuals with obesity.

## 5 CONCLUSION

In sum, this study demonstrated that early reductions in IWS predicted greater weight loss at week 46 of the intervention. Associations between early reductions in IWS and subsequent weight loss may inform the development of future weight management programs. Furthermore, these findings suggest that physical activity did not mediate the relationship between IWS and weight loss outcomes, highlighting the need for future research examining other potential explanations for this relationship. Overall, more comprehensive longitudinal research is needed to better understand the interrelationships among IWS, health behaviors, and weight‐related health outcomes.

## AUTHOR CONTRIBUTIONS

**Miriam Sheynblyum**: Conceptualisation; formal analysis; methodology; writing‐original draft. **Thomas A. Wadden**: Resources; supervision; writing‐review and editing. **Janet D. Latner**: Writing‐review and editing. **Rebecca L. Pearl**: Conceptualisation; funding acquisition; investigation; methodology; project administration; supervision; writing‐review and editing.

## CONFLICT OF INTEREST STATEMENT

The authors declare no conflicts of interest.

## STUDY PREREGISTRATION

https://osf.io/xgm26?view_only=8246f98768984f2999c78f13030b439d.

## Supporting information

Supporting Information S1
