# Supplementary material for: Longitudinal associations of internalized weight stigma with physical activity and weight loss
Source: Obes Sci Pract. 2024 Jul 3;10(4):e773. doi: 10.1002/osp4.773 (PMC11222290; doi:10.1002/osp4.773)
Supplement: Supplementary file 1 — Supporting Information S1 [file OSP4-10-e773-s001.docx]

**Longitudinal Associations of Internalized Weight Stigma with Physical Activity and Weight Loss**

Miriam Sheynblyum^1^, Thomas A. Wadden^2^, Janet D. Latner^3^, and Rebecca L. Pearl^1^

^1^Department of Clinical and Health Psychology, University of Florida, Gainesville, FL, USA

^2^Department of Psychiatry, Perelman School of Medicine at the University of Pennsylvania,

Philadelphia, PA, USA

^3^Department of Psychology, University of Hawaii at Manoa, Honolulu, HI, USA

**Online Supporting Information**

**Table S1** *Partial Correlations with the WBIS: Baseline-Week 20*

| Variable (change from baseline-week 20) | 1 |
| --- | --- |
| 1. Weight Bias Internalization Scale (WBIS) |  |
| 1. Weight Self-Stigma Questionnaire (WSSQ) | .326 ** |
| 1. Percent weight change | .208 |
| 1. Energy expenditure (kcal/week) ^a^ | .049 |
| 1. Unbouted moderate physical activity per day (minutes/day) | .022 |
| 1. Bouted moderate-to-vigorous physical activity minutes per day (MVPA; 20-minute bouts) ^b^ | .163 |

Note. Sample sizes for correlations were as follows: Variables 1 and 2: *n*=92; Variable 3: *n*=90; Variable 4: *n*=88; Variable 5: *n*=76; Variable 6: *n*=76.

Partial correlations control for treatment condition.

Average change values for untransformed variables (Mean ± Standard Deviation) were as follows: WBIS = -1.29 ± 0.93; WSSQ = -6.80 ± 7.17; Percent Weight Change= -6.07 ± 4.65; Energy Expenditure = 389.07 ± 859.14 kcal/week; Unbouted moderate physical activity = 9.13 ± 34.41 min/d; Bouted MVPA =10.21 ± 16.23 min/d.

^a^ Variable was transformed using the square-root method

^b^ Variable was transformed using the base-10 logarithm

* *p* < .05; ** indicates *p* < .01

**Table S2** *Partial Correlations with the WBIS: Baseline-Week 46*

| Variable (change from baseline-week 46) | 1 |
| --- | --- |
| 1. Weight Bias Internalization Scale (WBIS) |  |
| 1. Weight Self-Stigma Questionnaire (WSSQ) | .511** |
| 1. Percent weight change | .198 |
| 1. Energy expenditure (kcal/week) ^a^ | -.003 |
| 1. Unbouted moderate physical activity per day (minutes/day) | -.110 |
| 1. Bouted moderate-to-vigorous physical activity minutes per day (MVPA; 20-minute bouts) ^b^ | -.057 |

Note. Note. Sample sizes for correlations were as follows: Variables 1 and 3: *n*=90; Variable 2: *n*=89; Variable 4: *n*=88; Variable 5: *n*=59; Variable 6: *n*=59.

Partial correlations control for treatment condition.

Average change values for untransformed variables (Mean ± Standard Deviation) were as follows: WBIS = -1.42 ± 1.07; WSSQ = -6.94 ± 8.23; Percent Weight Change= -6.73 ± 6.93; Energy Expenditure = 186.55 ± 856.28 kcal/week; Unbouted moderate physical activity = -9.58 ± 43.88 min/d; Bouted MVPA = 10.19 ± 18.60 min/d.

^a^ Variable was transformed using the square-root method

^b^ Variable was transformed using the base-10 logarithm

* *p* < .05; ** indicates *p* < .01.

**Table S3** *Partial Correlation with the WBIS: Baseline-Week 72*

| Variable (change from baseline-week 72) | 1 |
| --- | --- |
| 1. Weight Bias Internalization Scale (WBIS) |  |
| 1. Weight Self-Stigma Questionnaire (WSSQ) | .492** |
| 1. Percent weight change | .205 |
| 1. Energy expenditure (kcal/week) ^a^ | .036 |
| 1. Unbouted moderate physical activity per day (minutes/day) | .042 |
| 1. Bouted moderate-to-vigorous physical activity minutes per day (MVPA; 20-minute bouts) ^b^ | -.103 |

Note. Sample sizes for correlations were as follows: Variables 1 and 2: *n*=85; Variable 3: *n*=89; Variable 4: *n*=83; Variable 5: *n*=71; Variable 6: *n*=71.

Partial correlations control for treatment condition.

Average change values for untransformed variables (Mean ± Standard Deviation) were as follows: WBIS = -1.60 ± 1.01; WSSQ = -8.29 ± 8.78; Percent Weight Change = -5.84 ± 8.16; Energy Expenditure = 390.05 ± 987.51 kcal/week; Unbouted moderate physical activity = -2.63 ± 45.54 min/d; Bouted MVPA = 8.87 ± 18.66 min/d.

^a^ Variable was transformed using the square-root method

^b^ Variable was transformed using the base-10 logarithm

* *p* < .05; ***p* < .01

**Table S4** *Bootstrap Mediation Results: Completer’s analysis for Baseline to Week 46 using the WBIS. Predictor: change in IWS from baseline to week 20; Mediator: change in physical activity from week 20 to 46; Outcome: percent weight loss (Model 1) or odds of ≥ 5% weight loss (Model 2) from baseline to week 46.*

| Model | Mediator | Path | Estimate | SE | CI |
| --- | --- | --- | --- | --- | --- |
| 1 | Unbouted | IWS -> unbouted moderate physical activity (*a*) | **-11.84** | **5.56** | **-23.44, -1.47** |
|  | moderate physical activity | Unbouted moderate physical activity -> percent weight change (*b*) | -0.02 | 0.02 | -0.06, 0.02 |
|  |  | Direct effect of IWS -> percent weight change (*c’*)  Total effect of IWS -> percent weight change (*c*) | 0.74  0.96 | 1.40  1.04 | -2.15, 3.21  -1.13, 3.06 |
|  |  | Indirect effect (*a x b*) | 0.22 | 0.27 | -0.23, 0.91 |
|  | Bouted MVPA | IWS -> Bouted MVPA (*a*) | -0.06 | 0.07 | -0.18, 0.09 |
|  |  | Bouted MVPA -> percent weight change (*b*) | -2.83 | 1.99 | -7.48, 0.51 |
|  |  | Direct effect of IWS -> percent weight change (*c’*)  Total effect of IWS -> percent weight change (*c*) | 0.79  0.96 | 1.27  1.04 | -1.93, 2.98  0.36, -1.13 |
|  |  | Indirect effect (*a x b*) | 0.17 | 0.25 | -0.19, 0.80 |
|  | Energy | IWS -> energy expenditure (*a*) | 83.71 | 106.89 | -126.97, 316.12 |
|  | expenditure | Energy expenditure -> percent weight change (*b*) | 0.00 | 0.00 | 0.00, 0.00 |
|  |  | Direct effect of IWS -> percent weight change (*c’*)  Total effect of IWS -> percent weight change (*c*) | 0.48  0.48 | 1.02  0.87 | -1.53, 2.39  -1.24, 2.21 |
|  |  | Indirect effect (*a x b*) | 0.01 | 0.11 | -0.26, 0.26 |
| 2 | Unbouted | IWS -> unbouted moderate physical activity (*a*) | **-11.84** | **5.59** | **-22.93, -1.79** |
|  | moderate physical activity | Unbouted moderate physical activity -> odds of ≥ 5% weight loss (*b*) | 0.01 | 0.01 | -0.01, 0.03 |
|  |  | Direct effect of IWS -> odds of ≥ 5% weight loss (*c’*) | -0.02 | 0.39 | -0.79, 0.85 |
|  |  | Indirect effect (*a x b*) | -0.11 | 0.14 | -0.49, 0.06 |
|  | Bouted MVPA | IWS -> Bouted MVPA (*a*) | -0.06 | 0.06 | -0.18, 0.07 |
|  |  | Bouted MVPA -> odds of ≥ 5% weight loss (*b*) | 0.33 | 0.88 | -1.16, 2.21 |
|  |  | Direct effect of IWS -> odds of ≥ 5% weight loss (*c’*) | -0.12 | 0.37 | -0.87, 0.62 |
|  |  | Indirect effect (*a x b*) | -0.02 | 0.09 | -0.29, 0.06 |
|  | Energy | IWS -> energy expenditure (*a*) | 83.71 | 104.91 | -112.22, 305.14 |
|  | expenditure | Energy expenditure -> odds of ≥ 5% weight loss (*b*) | 0.00 | 0.00 | 0.00, 0.00 |
|  |  | Direct effect of IWS -> odds of ≥ 5% weight loss (*c’*) | -0.25 | 0.31 | -0.94, 0.29 |
|  |  | Indirect effect (*a x b*) | 0.01 | 0.03 | -0.06, 0.09 |

Note. IWS = internalized weight stigma; MVPA = moderate-to-vigorous physical activity. This table contains unstandardized coefficients. Confidence intervals that do not contain zero indicate a significant model and are highlighted in bold.

**Table S5** *Bootstrap Mediation Results: Completer’s analysis for Baseline to Week 72 using the WBIS. Predictor: change in IWS from baseline to week 20; Mediator: change in physical activity from week 20 to 72; Outcome: percent weight loss (Model 3) or odds of ≥ 5% weight loss (Model 4) from baseline to week 72.*

| Model | Mediator | Path | Estimate | SE | 95% CI |
| --- | --- | --- | --- | --- | --- |
| 3 | Unbouted | IWS -> unbouted moderate physical activity (*a*) | -3.21 | 6.01 | -13.03, 10.53 |
|  | moderate physical activity | Unbouted moderate physical activity -> percent weight change (*b*) | -0.04 | 0.02 | -0.07, 0.00 |
|  |  | Direct effect of IWS -> percent weight change (*c’*)  Total effect of IWS -> percent weight change (*c*) | 0.20  0.33 | 1.49  1.22 | -2.83, 2.89  -2.11, 2.76 |
|  |  | Indirect effect (*a x b*) | 0.13 | 0.26 | -0.45, 0.68 |
|  | Bouted MVPA | IWS -> Bouted MVPA (*a*) | -0.02 | 0.09 | -0.17, 0.19 |
|  |  | Bouted MVPA -> percent weight change (*b*) | -0.80 | 1.63 | -4.27, 2.14 |
|  |  | Direct effect of IWS -> percent weight change (*c’*)  Total effect of IWS -> percent weight change (*c*) | 0.31  0.33 | 1.50  1.22 | -2.70, 2.80  0.79, -2.11 |
|  |  | Indirect effect (*a x b*) | 0.01 | 0.15 | -0.24, 0.42 |
|  | Energy | IWS -> energy expenditure (*a*) | -133.27 | 116.43 | -371.35, 97.70 |
|  | expenditure | Energy expenditure -> percent weight change (*b*) | 0.00 | 0.00 | 0.00, 0.00 |
|  |  | Direct effect of IWS -> percent weight change (*c’*)  Total effect of IWS -> percent weight change (*c*) | -0.34  -0.35 | 1.21  1.00 | -2.62, 2.08  -2.34, 1.65 |
|  |  | Indirect effect (*a x b*) | -0.01 | 0.18 | -0.25, 0.46 |
| 4 | Unbouted | IWS -> unbouted moderate physical activity (*a*) | -3.21 | 6.03 | -13.35, 10.81 |
|  | moderate physical activity | Unbouted moderate physical activity -> odds of ≥ 5% weight loss (*b*) | **0.01** | **0.01** | **0.00, 0.03** |
|  |  | Direct effect of IWS -> odds of ≥ 5% weight loss (*c’*) | -0.05 | 0.36 | -0.86, 0.66 |
|  |  | Indirect effect (*a x b*) | -0.05 | 0.11 | -0.24, 0.20 |
|  | Bouted MVPA | IWS -> Bouted MVPA (*a*) | -0.02 | 0.09 | -0.19, 0.16 |
|  |  | Bouted MVPA -> odds of ≥ 5% weight loss (*b*) | 0.31 | 0.42 | -0.43, 1.25 |
|  |  | Direct effect of IWS -> odds of ≥ 5% weight loss (*c’*) | -0.09 | 0.34 | -0.79, 0.56 |
|  |  | Indirect effect (*a x b*) | 0.00 | 0.04 | -0.12, 0.06 |
|  | Energy | IWS -> energy expenditure (*a*) | -133.27 | 113.23 | -353.55, 87.87 |
|  | expenditure | Energy expenditure -> odds of ≥ 5% weight loss (*b*) | 0.00 | 0.00 | 0.00, 0.00 |
|  |  | Direct effect of IWS -> odds of ≥ 5% weight loss (*c’*) | -0.11 | 0.29 | -0.66, 0.52 |
|  |  | Indirect effect (*a x b*) | 0.00 | 0.06 | -0.16, 0.07 |

Note. IWS = internalized weight stigma; MVPA = moderate-to-vigorous physical activity. This table contains unstandardized coefficients. Confidence intervals that do not contain zero indicate a significant model and are highlighted in bold.

**Table S6** *Bootstrap Mediation Results: LOCF analysis for Baseline to Week 46 using the WBIS. Predictor: change in IWS from baseline to week 20; Mediator: change in physical activity from week 20 to 46; Outcome: percent weight loss (Model 3) or odds of ≥ 5% weight loss (Model 2) from baseline to week 46.*

| Model | Mediator | Path | Estimate | SE | CI |
| --- | --- | --- | --- | --- | --- |
| 1 | Unbouted | IWS -> unbouted moderate physical activity (*a*) | -4.51 | 3.68 | -11.51, 2.38 |
|  | moderate physical activity | Unbouted moderate physical activity -> percent weight change (*b*) | 0.00 | 0.02 | -0.02, 0.05 |
|  |  | Direct effect of IWS -> percent weight change (*c’*)  Total effect of IWS -> percent weight change (*c*) | **1.94**  **1.92** | **0.72**  **0.70** | **0.57, 3.36**  **0.01, 0.53** |
|  |  | Indirect effect (*a x b*) | -0.02 | 0.11 | -0.31, 0.14 |
|  | Bouted MVPA | IWS -> Bouted MVPA (*a*) | -0.05 | 0.04 | -0.13, 0.03 |
|  |  | Bouted MVPA -> percent weight change (*b*) | -1.50 | 1.69 | -5.28, 1.48 |
|  |  | Direct effect of IWS -> percent weight change (*c’*)  Total effect of IWS -> percent weight change (*c*) | **1.84**  **1.92** | **0.71**  **0.70** | **0.47, 3.23**  **0.01, 0.53** |
|  |  | Indirect effect (*a x b*) | 0.08 | 0.11 | -0.11, 0.32 |
|  | Energy | IWS -> energy expenditure (*a*) | 87.31 | 75.17 | -64.48, 253.66 |
|  | expenditure | Energy expenditure -> percent weight change (*b*) | 0.00 | 0.00 | 0.00, 0.00 |
|  |  | Direct effect of IWS -> percent weight change (*c’*)  Total effect of IWS -> percent weight change (*c*) | **1.74**  **1.79** | **0.72**  **0.67** | **0.30, 3.19**  **0.45, 3.12** |
|  |  | Indirect effect (*a x b*) | 0.04 | 0.10 | -0.17, 0.27 |
| 2 | Unbouted | IWS -> unbouted moderate physical activity (*a*) | -4.51 | 3.70 | -11.67, 2.58 |
|  | moderate physical activity | Unbouted moderate physical activity -> odds of ≥ 5% weight loss (*b*) | 0.00 | 0.01 | -0.02, 0.02 |
|  |  | Direct effect of IWS -> odds of ≥ 5% weight loss (*c’*) | **-0.63** | **0.27** | **-1.22, -0.19** |
|  |  | Indirect effect (*a x b*) | -0.01 | 0.05 | -0.12, 0.08 |
|  | Bouted MVPA | IWS -> Bouted MVPA (*a*) | -0.05 | 0.04 | -0.13, 0.03 |
|  |  | Bouted MVPA -> odds of ≥ 5% weight loss (*b*) | 0.25 | 0.73 | -1.18, 1.84 |
|  |  | Direct effect of IWS -> odds of ≥ 5% weight loss (*c’*) | **-0.64** | **0.27** | **-1.25, -0.18** |
|  |  | Indirect effect (*a x b*) | -0.01 | 0.05 | -0.15, 0.04 |
|  | Energy | IWS -> energy expenditure (*a*) | 87.31 | 71.72 | -57.15, 230.01 |
|  | expenditure | Energy expenditure -> odds of ≥ 5% weight loss (*b*) | 0.00 | 0.00 | 0.00, 0.00 |
|  |  | Direct effect of IWS -> odds of ≥ 5% weight loss (*c’*) | **-0.61** | **0.26** | **-1.17, -0.18** |
|  |  | Indirect effect (*a x b*) | 0.00 | 0.03 | -0.07, 0.05 |

Note. LOCF = last observation carried forward; IWS = internalized weight stigma; MVPA = moderate-to-vigorous physical activity.

This table contains unstandardized coefficients. Confidence intervals that do not contain zero indicate a significant model and are highlighted in bold.

**Table S7** *Bootstrap Mediation Results: LOCF analysis for Baseline to Week 72 using the WBIS. Predictor: change in IWS from baseline to week 20; Mediator: change in physical activity from week 20 to 72; Outcome: percent weight loss (Model 3) or odds of ≥ 5% weight loss (Model 4) from baseline to week 72.*

| Model | Mediator | Path | Estimate | SE | CI |
| --- | --- | --- | --- | --- | --- |
| 3 | Unbouted | IWS -> unbouted moderate physical activity (*a*) | 1.76 | 3.96 | -6.03, 9.79 |
|  | moderate physical activity | Unbouted moderate physical activity -> percent weight change (*b*) | -0.03 | 0.02 | -0.07, 0.00 |
|  |  | Direct effect of IWS -> percent weight change (*c’*)  Total effect of IWS -> percent weight change (*c*) | 0.74  0.68 | 0.92  0.82 | -1.08, 2.62  0.41, -0.94 |
|  |  | Indirect effect (*a x b*) | -0.06 | 0.15 | -0.40, 0.23 |
|  | Bouted MVPA | IWS -> Bouted MVPA (*a*) | 0.00 | 0.07 | -0.14, 0.15 |
|  |  | Bouted MVPA -> percent weight change (*b*) | -0.89 | 1.20 | -3.12, 1.49 |
|  |  | Direct effect of IWS -> percent weight change (*c’*)  Total effect of IWS -> percent weight change (*c*) | 0.67  0.68 | 0.91  0.82 | -1.02, 2.50  0.41, -0.94 |
|  |  | Indirect effect (*a x b*) | 0.00 | 0.10 | -0.26, 0.20 |
|  | Energy | IWS -> energy expenditure (*a*) | -106.56 | 96.61 | -298.70, 66.80 |
|  | expenditure | Energy expenditure -> percent weight change (*b*) | 0.00 | 0.00 | 0.00, 0.00 |
|  |  | Direct effect of IWS -> percent weight change (*c’*)  Total effect of IWS -> percent weight change (*c*) | 0.50  0.50 | 0.95  0.80 | -1.33, 2.33  -1.09, 2.09 |
|  |  | Indirect effect (*a x b*) | 0.00 | 0.11 | -0.17, 0.29 |
| 4 | Unbouted | IWS -> Unbouted moderate physical activity (*a*) | 1.76 | 4.17 | -6.10, 10.38 |
|  | moderate physical activity | Unbouted moderate physical activity -> odds of ≥ 5% weight loss (*b*) | 0.01 | 0.01 | 0.00, 0.03 |
|  |  | Direct effect of IWS -> odds of ≥ 5% weight loss (*c’*) | -0.30 | 0.26 | -0.85, 0.18 |
|  |  | Indirect effect (*a x b*) | 0.02 | 0.05 | -0.08, 0.17 |
|  | Bouted MVPA | IWS -> Bouted MVPA (*a*) | 0.00 | 0.07 | -0.13, 0.14 |
|  |  | Bouted MVPA -> odds of ≥ 5% weight loss (*b*) | 0.44 | 0.36 | -0.20, 1.23 |
|  |  | Direct effect of IWS -> odds of ≥ 5% weight loss (*c’*) | -0.28 | 0.23 | -0.78, 0.13 |
|  |  | Indirect effect (*a x b*) | 0.00 | 0.04 | -0.09, 0.09 |
|  | Energy | IWS -> energy expenditure (*a*) | -106.56 | 95.34 | -301.51, 76.78 |
|  | expenditure | Energy expenditure -> odds of ≥ 5% weight loss (*b*) | 0.00 | 0.00 | 0.00, 0.00 |
|  |  | Direct effect of IWS -> odds of ≥ 5% weight loss (*c’*) | -0.29 | 0.23 | -0.73, 0.16 |
|  |  | Indirect effect (*a x b*) | 0.00 | 0.03 | -0.09, 0.06 |

Note. LOCF = last observation carried forward; IWS = internalized weight stigma; MVPA = moderate-to-vigorous physical activity. This table contains unstandardized coefficients.

| **Table S8** *Bootstrap Mediation Results: Sensitivity Completer’s Analysis for Baseline to Week 46 (Models 1 and 2) and Baseline to Week 72 (Models 3 and 4) using the WSSQ* | | | | | |
| --- | --- | --- | --- | --- | --- |
| Model | Mediator | Path | Estimate | SE | CI |
| 1 | Unbouted | Indirect effect (*a x b*) | 0.00 | 0.01 | -0.12, 0.01 |
|  | moderate | IWS -> unbouted moderate physical activity (*a*) | 0.13 | 1.14 | -1.81, 2.79 |
|  | physical activity | Unbouted moderate physical activity -> percent weight change (*b*) | -0.02 | 0.02 | -0.06, 0.02 |
|  |  | Direct effect of IWS -> percent weight change (*c’*)  Total effect of IWS -> percent weight change (*c*) | 0.18  0.18 | 0.13  0.13 | -0.03, 0.50  0.17, -0.08 |
|  |  | Indirect effect (*a x b*) | 0.00 | 0.04 | -0.09, 0.08 |
|  | Bouted MVPA | IWS -> Bouted MVPA (*a*) | -0.01 | 0.01 | -0.02, 0.00 |
|  |  | Bouted MVPA -> percent weight change (*b*) | -2.54 | 2.14 | -7.56, 0.93 |
|  |  | Direct effect of IWS -> percent weight change (*c’*)  Total effect of IWS -> percent weight change (*c*) | 0.15  0.18 | 0.13  0.13 | -0.06, 0.44  0.17, -0.08 |
|  |  | Indirect effect (*a x b*) | 0.03 | 0.03 | -0.01, 0.10 |
|  | Energy | IWS -> energy expenditure (*a*) | -21.23 | 15.75 | -52.87, 9.50 |
|  | expenditure | Energy expenditure -> percent weight change (*b*) | 0.00 | 0.00 | 0.00, 0.00 |
|  |  | Direct effect of IWS -> percent weight change (*c’*)  Total effect of IWS -> percent weight change (*c*) | 0.17  0.16 | 0.11  0.11 | -0.02, 0.41  0.14, -0.06 |
|  |  | Indirect effect (*a x b*) | -0.01 | 0.02 | -0.06, 0.03 |
| 2 | Unbouted | IWS -> unbouted moderate physical activity (*a*) | 0.13 | 1.09 | -1.74, 2.42 |
|  | moderate physical activity | Unbouted moderate physical activity -> odds of ≥ 5% weight loss (*b*) | 0.01 | 0.01 | -0.01, 0.03 |
|  |  | Direct effect of IWS -> odds of ≥ 5% weight loss (*c’*) | -0.04 | 0.05 | -0.16, 0.05 |
|  |  | Indirect effect (*a x b*) | 0.00 | 0.02 | -0.03, 0.04 |
|  | Bouted MVPA | IWS -> Bouted MVPA (*a*) | -0.01 | 0.01 | -0.02, 0.00 |
|  |  | Bouted MVPA -> odds of ≥ 5% weight loss (*b*) | 0.26 | 0.89 | -1.32, 2.26 |
|  |  | Direct effect of IWS -> odds of ≥ 5% weight loss (*c’*) | -0.03 | 0.05 | -0.16, 0.05 |
|  |  | Indirect effect (*a x b*) | 0.00 | 0.01 | -0.03, 0.02 |
|  | Energy | IWS -> energy expenditure (*a*) | -21.23 | 16.13 | -53.25, 10.26 |
|  | expenditure | Energy expenditure -> odds of ≥ 5% weight loss (*b*) | 0.00 | 0.00 | 0.00, 0.00 |
|  |  | Direct effect of IWS -> odds of ≥ 5% weight loss (*c’*) | -0.05 | 0.04 | -0.14, 0.01 |
|  |  | Indirect effect (*a x b*) | 0.00 | 0.01 | -0.02, 0.01 |
| 3 | Unbouted | IWS -> unbouted moderate physical activity (a) | -0.66 | 0.78 | -2.35, 0.80 |
|  | moderate physical activity | Unbouted moderate physical activity -> percent weight change (*b*) | -0.04 | 0.02 | -0.07, 0.01 |
|  |  | Direct effect of IWS -> percent weight change (*c’*)  Total effect of IWS -> percent weight change (*c*) | 0.24  0.26 | 0.18  0.15 | -0.05, 0.66  0.09, -0.04 |
|  |  | Indirect effect (*a x b*) | 0.02 | 0.03 | -0.04, 0.09 |
|  | Bouted MVPA | IWS -> Bouted MVPA (*a*) | 0.02 | 0.01 | 0.00, 0.04 |
|  |  | Bouted MVPA -> percent weight change (*b*) | -1.32 | 1.51 | -4.69, 1.32 |
|  |  | Direct effect of IWS -> percent weight change (*c’*)  Total effect of IWS -> percent weight change (*c*) | 0.28  0.26 | 0.18  0.15 | -0.01, 0.69  0.09, -0.04 |
|  |  | Indirect effect (*a x b*) | -0.02 | 0.03 | -0.08, 0.03 |
|  | Energy | IWS -> energy expenditure (*a*) | -15.90 | 14.47 | -48.10, 10.18 |
|  | expenditure | Energy expenditure -> percent weight change (*b*) | 0.00 | 0.00 | 0.00, 0.00 |
|  |  | Direct effect of IWS -> percent weight change (*c’*)  Total effect of IWS -> percent weight change (*c*) | 0.15  0.14 | 0.13  0.12 | -0.10, 0.42  0.25, -0.10 |
|  |  | Indirect effect (*a x* *b*) | 0.00 | 0.02 | -0.03, 0.07 |
| 4 | Unbouted | IWS -> unbouted moderate physical activity (*a*) | -0.66 | 0.81 | -2.45, 0.80 |
|  | moderate physical activity | Unbouted moderate physical activity -> odds of ≥ 5% weight loss (*b*) | **0.01** | **0.01** | **0.00, 0.03** |
|  |  | Direct effect of IWS -> odds of ≥ 5% weight loss (c’) | -0.05 | 0.06 | -0.19, 0.03 |
|  |  | Indirect effect (*a* *x* *b*) | -0.01 | 0.01 | -0.04, 0.01 |
|  | Bouted MVPA | IWS -> Bouted MVPA (*a*) | 0.02 | 0.01 | 0.00, 0.04 |
|  |  | Bouted MVPA -> odds of ≥ 5% weight loss (*b*) | 0.42 | 0.43 | -0.31, 1.39 |
|  |  | Direct effect of IWS -> odds of ≥ 5% weight loss (*c’*) | -0.06 | 0.05 | -0.19, 0.01 |
|  |  | Indirect effect (*a x b*) | 0.01 | 0.01 | -0.01, 0.03 |
|  | Energy | IWS -> energy expenditure (*a*) | -15.90 | 14.48 | -47.94, 9.54 |
|  | expenditure | Energy expenditure -> odds of ≥ 5% weight loss (*b*) | 0.00 | 0.00 | 0.00, 0.00 |
|  |  | Direct effect of IWS -> odds of ≥ 5% weight loss (*c’*)  Indirect effect *(a x b)* | -0.03  0.00 | 0.04  0.01 | -0.12, 0.03  -0.02, 0.01 |

Note. WSSQ = Weight Self-Stigma Questionnaire; IWS = internalized weight stigma; MVPA = moderate-to-vigorous physical activity. This table contains unstandardized coefficients. Confidence intervals that do not contain zero indicate a significant model and are highlighted in bold.
